# “We need more big trees as well as the grass roots”: going beyond research capacity building to develop sustainable careers in mental health research in African countries

**DOI:** 10.1186/s13033-020-00388-1

**Published:** 2020-08-14

**Authors:** Lisa F. Langhaug, Helen Jack, Charlotte Hanlon, Stefan Holzer, Katherine Sorsdahl, Barbara Mutedzi, Walter Mangezi, Christopher Merritt, Atalay Alem, Robert Stewart, Chiwoza Bandawe, Rosemary Musesengwa, Melanie Abas, Dixon Chibanda, Crick Lund

**Affiliations:** 1 African Mental Health Research Initiative (AMARI), Department of Psychiatry, College of Health Sciences, University of Zimbabwe, Harare, Zimbabwe; 2 Centre for Global Mental Health, Health Service and Population Research Department, Institute of Psychiatry, Psychology and Neuroscience, Kings College London, London, UK; 3 Department of Medicine, University of Washington, Seattle, WA USA; 4 Department of Psychiatry, WHO Collaborating Centre in Mental Health Research and Capacity-Building, School of Medicine, College of Health Sciences, Addis Ababa University, Addis Ababa, Ethiopia; 5 Centre for Innovative Drug Development and Therapeutic Trials for Africa (CDT-Africa), College of Health Sciences, Addis Ababa University, Addis Ababa, Ethiopia; 6 Department of Mental Health, College of Medicine, University of Malawi, Blantyre, Malawi; 7 Alan J. Flisher Centre for Public Mental Health, Department of Psychiatry and Mental Health, University of Cape Town, Cape Town, South Africa

**Keywords:** Global mental health, Africa, Research Policy, Research funding, Research capacity strengthening

## Abstract

**Background:**

There are substantial gaps in our knowledge regarding the aetiology of mental, neurological and substance use disorders in sub-Saharan Africa, and the cost-effectiveness and scalability of interventions to reduce the burden of these conditions on the continent. To address these gaps, international investment has focussed on building research capacity, including funding doctoral students in African countries, to support development of high quality, contextually relevant interventions. Absent, however, is an understanding of how capacity building feeds into research careers.

**Methods:**

Within a broader mental health research capacity-building initiative (African Mental Health Research Initiative), we conducted 52 qualitative interviews with early-career researchers, policymakers, academics, and service users from four African countries (Ethiopia, Malawi, South Africa, and Zimbabwe) and with international funders of mental health research. The interview guide focused on the research context, planning, and priorities and how respondents perceive research careers and funding. Thematic analysis was applied to the transcribed interviews.

**Results:**

Five components of a research career emerged: (i) research positions; (ii) research skills; (iii) funding; (iv) research commitment from African countries; and (v) advocacy. All stakeholders wanted more high-impact African researchers, but few saw a clear, replicable track for developing their careers within universities or their Ministries of Health in their African countries. This stemmed, in part, from the lack of support for infrastructure that enables high-quality research: grants administration, mentorship, university leadership, research culture, and open communication between policymakers and researchers.

**Conclusions:**

This study highlights the importance of developing research infrastructure alongside capacity-building efforts. International funders should invest in grant management at African universities which would place them at the centre of research initiatives. African universities should prioritise the creation of a research culture by developing and promoting well-defined research tracks for both clinicians and academics, investing in grant management, and raising the profile of research within their institutions.

## Key messages

- Researchers from African countries should take a leading role in international research collaborations to address inherently inequitable relationships.
- Governments of African countries should invest funds and human resources in mental health research, enabling development of evidence-based policies to reduce research and treatment gaps.
- Specific research career pathways in mental health research need to be fashioned in order to retain junior researchers in their home countries and become future leaders.

## Background

Globally, mental, neurological, and substance use (MNS) disorders are the leading cause of health disability [1] and in the most recent Global Burden of Disease Study 2017, mental and substance use disorders accounted for 6.7% of the global disease burden [2]. This remains critical as mental health conditions impact not only most of the major health problems including HIV, maternal and child health, adolescent sexual health, and many non-communicable diseases (diabetes, obesity, cardiovascular diseases) but also reaches beyond that, playing a key role when addressing human rights abuses, DALYS, and mortality [3]. Despite this high burden of disease, and the fact that 85% of the worlds’ population lives in LMICs, research capacity in mental health is generally limited, particularly in sub-Saharan Africa. This results in an even larger gap in MNS research, particularly research on interventions and care delivery systems [4]. Globally, only 1% of mental health research has been conducted in all low-income countries and 10% in middle-income countries [5], with 80% of this research coming from South Africa. Collaborative authorship also reflects this bias: when publications concerning health in sub-Saharan Africa include authors from the US, Canada, and Europe, only 23% of first authors are from the country of focus, and 13.5% of papers have no local co-authors [6].

Many factors contribute to the lack of mental health research in Africa, including few opportunities for training and mentorship at African institutions; lack of integration of mental health into general medical settings, which limits opportunities for funding and interdisciplinary collaboration with other, better supported fields such as HIV and maternal and child health; minimal funding and institutional support; and the lack of a critical mass of MNS researchers and leaders [5, 7–9]. The small number of researchers, limited training opportunities for researchers, and lack of journals from African countries makes it difficult for their voices to be present in the international academic discourse on mental health [10]. It is particularly important for their voices to be part of this discourse because of the important sociocultural and contextual expressions of mental distress and illness [11]. International consensus on mental health research priorities agrees that these context-specific factors should inform intervention development and implementation [12].

In recent years, a number of mental health research capacity-building programs have been implemented in Africa to address this gap. These programs have worked to build the clinical and research capacity of medical school departments, including a psychiatry department in Zimbabwe [13–15]; provided small grants, external supervision and mentorship for LMIC PhD students within ongoing trials and projects [4, 16, 17]; and supported post-graduate programmes in African countries: for example, the MPhil in Public Mental at the University of Cape Town and PhD programme in mental health epidemiology at Addis Ababa University. Other linked research programmes have included a strong capacity building component, for example the Africa Focus on Intervention Research for Mental health (AFFIRM) [16, 18, 19], Emerging Mental health systems in low and middle-income countries (EMERALD) [4], Partnership for Mental health Development in sub-Saharan Africa (PaM-D) [20] and the Programme for Improving Mental health carE (PRIME) [17]. Most recently, the African Mental Health Research Initiative (AMARI) has, as its primary focus, the support of postgraduate researchers in four African countries: Ethiopia, Malawi, South Africa, and Zimbabwe [21].

These programs have primarily focused on training in research methods, external supervision and dissemination of findings and have led to the growth of academic departments that focus on mental health. However, it has been well documented that recruitment and retention of healthcare professionals in LMICs is challenging given the resource-constrained environments and opportunities to find work in HICs [13, 22, 23]. Qualitative studies on recruitment and retention of mental health workers (primarily those who provide clinical care) in Ghana [24] and Zimbabwe [13] have highlighted the importance of opportunities for ongoing professional development and career advancement. Despite the attention to building research capacity in mental health, there has been less focus on creating career pathways that support researchers to remain in low-resource settings.

Accordingly, the aim of this study was to explore research priorities, the funding environment, and career pathways for MNS researchers across four African countries (Ethiopia, Malawi, South Africa and Zimbabwe) participating in AMARI (https://amari-africa.org/).

## Methods

### Programme and setting

The African Mental Health Research Initiative (AMARI) is a collaboration among four African universities (Addis Ababa University, University of Cape Town, University of Malawi College of Medicine, and University of Zimbabwe College of Health Sciences) and universities in the United Kingdom (King’s College London, the London School of Hygiene and Tropical Medicine, and the Liverpool School of Tropical Medicine) that aims to support the development of high-calibre MNS researchers who conduct research that meets the needs of the four host countries: Ethiopia, Malawi, South Africa, and Zimbabwe. AMARI is recruiting and training 48 MNS research fellows at master’s, PhD and post-doctorate levels, with the intent of equipping them with the necessary research, teaching and leadership skills to build a viable and sustainable research network in the African region. With financial support from AMARI, fellows enroll in a PhD or master’s program at an African university. Fellows have local primary supervisors with some receiving additional support from international supervisors. Fellows attend short courses on research methods, research career development, developmental mentoring and academic writing that may not be available at their home institutions [21]. AMARI is building capacity to deliver these courses within each country for the ongoing development of research and leadership skills.

While a detailed brief on each university can be found in the Additional file 1: Appendix A, there are some important features to highlight concerning previous experience with research capacity building partnerships between the universities involved in AMARI. All four universities collaborated in AFFIRM, a research and capacity building initiative that supported MPhil fellowships, funding four PhD students nested within AFFIRM trials, and short courses in specialist research skills [16, 18]. The universities in South Africa and Ethiopia were part of PRIME, a LMIC-led partnership which provided research evidence for the development, implementation and scaling up of integrated district mental healthcare plans in five countries with 21 PhD students nested within PRIME [25]. These two universities, together with the University of Ibadan and Makerere University, were also involved in EMERALD (Emerging mental health systems in low- and middle-income countries), which also included capacity building for emerging mental health researchers and supported nine PhD students from LMICs, six of whom were from Africa [4]. The University of Cape Town and Stellenbosch Universities also host a master’s course in public mental health that recruits students internationally, and specifically from all across Africa. In addition, the Centre for Global Mental Health at King’s College London enjoys a bidirectional capacity-building relationship, with Addis Ababa University in Ethiopia contributing to lectures and hosting of MSc students in Ethiopia. Since 2011, Addis Ababa University has run a PhD programme in mental health epidemiology. Prior to the start of AMARI, partners from King’s College London and Liverpool have had long-established research and capacity building partnerships of over 10 years with the University of Zimbabwe [14].

### Study design and sampling

We conducted a qualitative study, comprising semi-structured interviews with key informants. Authors used their position as international and national leaders in mental health (CH, AA, DC, WM, RS, CB, SH, KS, CL) to purposively select participants who represented influential stakeholders in the mental health field in each of the four countries and included leaders from academia, government, clinical practitioners, service users, and AMARI fellows. Participants and interviewers were of both genders. All fellows were included if they were registered for a PhD or a Post-Doctoral fellowship with AMARI and had been a part of the programme for at least 2 years. These fellows were felt to be further along in their research careers than the MPhils students. Country teams used their contextual knowledge to select participants with expertise and experience who could offer a broad range of perspectives. Chain referral sampling was used to obtain additional contributors [26]. Participants were then invited to take part in the study. All interviewers (SH, CH, WM, HJ, KS, CM) were experienced in qualitative interviewing. Within the four countries, interviewers were familiar with the stakeholders they engaged. However, AMARI fellows were interviewed by someone external to the programme (BM). In addition, leading international funding organisations that support global health research were approached to take part in the interviews (CM, HJ). Both in-country and international participants were included because partnerships between local and international stakeholders are likely to be important for ongoing development of MNS research capacity in the region.

### Data collection

AMARI investigators conducted all interviews in English using semi-structured interview guides that focused on how research priorities are set, how research is funded, research careers, the relationship between academia and government, and challenges that early-career researchers face. On average, each interview took about an hour. The interview guide was modified for each group: (1) international funders, (2) LMIC academics and policymakers, and (3) AMARI fellows (see Additional file 2: Appendix B for a sample guide). Interviews were conducted privately either in-person or via Skype and no repeat visits to participants were deemed necessary. All interviews were recorded, transcribed by an independent contractor, and then checked by the interviewers for clarity. As the transcripts were clear, participants were not asked to review them.

### Data analysis

Two independent researchers (LL, BM) applied thematic analysis to the interviews [27]. One of them (LL) is a PhD supervisor for some of the AMARI fellows; the second (BM) has a background in medical anthropology. This combination allowed for both an external and internal lens to be employed during analysis. Following transcription, one author (LL) read through the transcripts in their entirety. Three randomly selected transcripts were then read and inductively assigned thematic codes. LL and BM then compared their independently developed code lists to create a joint code sheet which was used to code the remaining transcripts. During coding, which was done manually, three new codes were added; transcripts that had been coded previously were re-read to see if these codes were present. Following analysis, it was agreed that theoretical data saturation had been reached [28].

Ethical approval was obtained from the Institutional Review Board of the College of Health Sciences, Addis Ababa University (Ethiopia), the College of Medicine Research and Ethics Committee (COMREC) in Malawi, the Human Research Ethics Committee (HREC), Faculty of Health Sciences at the University of Cape Town (South Africa), the Medical Research Council of Zimbabwe (Zimbabwe), and the Research Ethics Committee (REC) at King’s College London. Written informed consent was obtained from all participants before the interview took place.

## Results

**Fig. 1:**
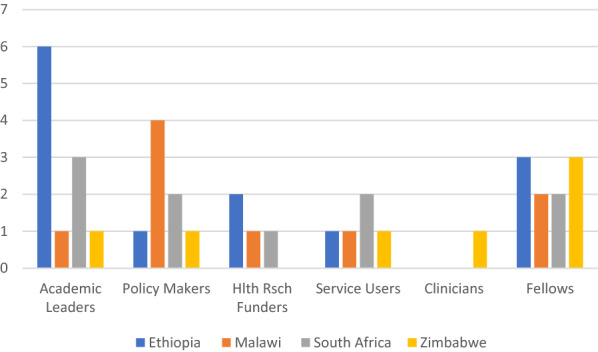
In-country respondents by profession

In total, 46 interviews were conducted with academic leaders, policymakers, clinicians, AMARI fellows, and service users from across the four countries (see Fig. 1); two AMARI fellows did not respond to the invitation for an interview (Malawi and Ethiopia). Of the 12 international funding organizations approached, six agreed to be interviewed. Three of them did not respond to the invitation and three others did not feel they had anyone who could discuss mental health.

Five components of a research career emerged from the coding process: (i) research positions; (ii) research skills; (iii) funding; (iv) LMIC research commitment; and (v) advocacy. Table 1 lists additional key quotes that support these thematic areas.

**Table 1:** Additional qualitative data from respondents

1. Research positions: Landscape for finding jobs to conduct research	
1.1	“Basically its stigma and discrimination on both the health care workers as well as the patients themselves… because you know what,… the fear of … being stigmatised in the clinics or community will actually hamper research…even as a researcher, if you are working in the field [of mental health] you are assumed to also suffer from the issue.”(8, programme Fellow)
1.2a	“It’s a very fragile pathway. (programme Fellow 4)
1.2b	“…because of the current socio-economic situation, the university has not been recruiting an optimum number of lecturers. So, I am sure even for those posts that would be available, … it may be difficult for the system to actually absorb all [the] PhD graduates.” (programme Fellow 9)
1.2c	“And it seems as if it’s mostly academic work or lectureship or lecturing… there isn’t a strict or set out career pathway for someone who would be interested in, in lots of research… (programme Fellow 10)
1.3	“…it will always be in conflict with my colleagues because people will say, ‘no, he is too busy with research but he is not doing his clinical work,’ and the HR [human resources staff] will be on my neck to say, ‘you were employed as a clinician; what are you doing?’” (programme Fellow 6)
1.4	“No, l am not aware of part time jobs but actually there is, ah, regulation that you can be hired like for 50% or 70% or 30%, but l don’t think we are using it properly. Maybe people don’t know or we are not encouraging it.” (academic leader 5, Ethiopia)
1.5a	“I think to start with here may be need to academia and policy makers to work hand in hand to actually develop a national research strategy for MNS” (programme Fellow 9)
1.5b	“I see the Ministry of Health is really operational and with a lot of service delivery and I don’t think there is much space for research funding within that structure.” (programme Fellow 4)
2. Research skills: developing the necessary skills to get research jobs, with focus on the need for mentorship	
2.1	“How do l build that perimeter of support so that l can train people who can really develop into highly skilled researchers in an African setting with knowledge of artillery resources, with knowledge of working in low income settings, … working with complicated systems or non-existing systems. But the quality has to be good, because they are going to be the generation who will train the next lot, and [so on]” (academic leader 2, South Africa)
2.2	“Instead of developing the talents that l have as an individual, it’s like, ‘you have to do what l have done because it has worked for me.’ Instead of me spreading my own wings l can’t do that because they are like clipped all the time. ….And most of these other supervisors, it’s like they don’t want you to grow bigger than them.” (programme Fellow 8)
2.3	“very few people for instance learn how to supervise and be supervised, how to be mentors.” (academic leader 2, South Africa)
2.4	“l don’t think everybody is cut out to be necessarily a good mentor, l think that supervision and mentoring skills are two different things so sometimes a very highly rated researcher might be excellent in terms of their academic content and strategic inputs but would not have the time or the hours to sit with a student and help mentor them and build capacity and check their analysis and assess them in a really practical level.” (policy maker 1, South Africa)
2.5	“Advocate, advocate and talk about it and… join it with other areas. Like…for women’s mental health, we can join it with the department of maternal and child health. And for adolescents, it can be family health. You can actually think of mental health in the HIV and AIDS [programmes]. those departments they [the researchers], they find it lucrative….because they get paid somehow.”(programme Fellow 8)
2.6a	“Even opportunities to travel and disseminate your research findings … because in that case then you are also able to develop other networking partnerships and also learn a lot.” (programme Fellow 5)
2.6b	“for me I think it was based on the network that I had within [the programme], to also get mentoring from seasoned researchers. …Because …we have had time to interact with more senior researchers..and they have pointed out funding opportunities. And that has then stimulated my interest in also looking for funding opportunities… But I would say the momentum was through the senior [researchers] across the [programme] Consortium. (programme Fellow 9)
3. Funding: discussions about challenges that LMIC researchers face in accessing research funding	
3.1	“It’s amazing to see how they [a UK university] can pull together a team and bring in an extra consultant before you have even got the grant money to help put this huge grant together…” (programme Fellow 4)
3.2	“if it’s a really small [LMIC] institution, they don’t have the disposable income to be paid in arrears. So they want payment up front, and [we are] very resistant to doing that because of the risks involved.” (programme officer, international funding agency 2)
4. Research commitment from the four African countries: how research is positioned within universities and ministries of health	
4.1	“I think… in country governments have a responsibility to provide a strong university infrastructure through which we can fund great scientists and great science… and then we can then fund those academics to do the science part of their job and not the teaching part of their job.” (international funding agency F4, head of section)
4.2	“But I think there’s also a lot of local responsibility, um, for building research capacity. Because like you can build the capacity of, you know, one individual to use a really sophisticated, I don’t know, statistical program. But if the university that they’re at doesn’t have a license for it, it doesn’t really make sense.” (international funding agency F1, programme officer,)
4.3	“But at the same time, we’ve got to make sure, that the stakeholders at both the government and the university levels in-country, are interested in providing that pathway, and working on developing that pathway for the researchers …Because, they’re *not* going to stay, if it’s not there.” (international funding agency F5, head of section)
4.4	“I think if our government is serious about research [and] research capacity, they also need to review the size of their funding.” (academic leader 2, South Africa)
4.5	“I think the lack of dedicated research funding at a local level, I think without having funds dedicated nationally to advance research in any area including mental health then very few people will go into those areas, because then people do not know what the future will be like..” (academic leader 1, Malawi)
4.6	“Government has set a priority to get 2000 [PhDs]. You have to invest in infrastructure so you recruit people to do PHDs and then if you don’t have the infrastructure you don’t produce quality. So if you just recruit people and give them PHDs that’s not going to help in terms of quality of the training.” (academic leader 5, Ethiopia)
5. Advocacy: perspectives on the connection between research and policy	
5.1	“Because you can’t just go and talk about the national budget, you can’t just go and say, ‘No, no, we should fund mental health.’ They will ask you, ‘Is it a problem? How big is the problem? And why should we worry about the problem?’ So we want to have…facts.” (MOH researcher 2, Malawi)
5.2a	“l think the researchers do communicate their work, but my issue is more on the messaging: what exactly is being said, how it is being said, how its packaged.” (policy maker 1, South Africa)
5.2b	“We need to put a human face, we need to explain it using a human story. For instance, a child having epilepsy, seizures, mild fits starting at the age of 2 years. With $1 per day treatment, no [more] fits or seizures. [She] can live a normal life without the attendant stigma, without the attendant loss of school hours and stuff like that. And more importantly, without attendant injuries and other complications of seizure disorder. If you put all those together for 2 years, 3 years how much is that: it’s less than less than $200. I get a child that has less complications, normal IQ, [and] productive [in] society.” (in-country rep for international funder 2, Ethiopia)
5.3	“l would want a situation whereby right from the beginning when you are putting in your proposal you are actually involving the policy makers.” (programme Fellow 8)
5.4	“[Research] is now more relevant than it was. When we look at the drafts of the current health sector strategic plan there is a lot more of it now that’s based on national research.” (MOH policy maker 1, Malawi)
5.5	“We don’t have a lot of resources, we have to choose the interventions which are formed by robust evidence, not just formal expert opinion” (MOH policy maker 4, Malawi)
5.6	“We managed to use data from a youth depression programme as a way of advocating for the inclusion of mental health into the school health programme.” (MOH policy maker 3, Malawi)
5.7	“From the different researches [conducted], we finally came up with an alcohol policy which is now at a level for approval. And also… studies done on tobacco…helped us to come up with a strategy.” (policy maker 3, Zimbabwe)
5.8	“[when] integrating mental health, it was found out that epilepsy was [one of them most common] disorders to be seen at the health centres….So … by availing the necessary drugs, by training the health professionals at health centres, and also making sure that we keep a track of them….this service has been integrated into primary health care in different areas. So, really knowing the magnitude of disease, the burden of disease really..set up policy so that we can … intervene in the right approach.” (policy maker 1, Ethiopia)

### Research positions

All AMARI PhD Fellows were keen to pursue research careers, but many struggled to see how they could achieve this. “*It’s a new area in our country. People haven’t had careers in research.”* (AMARI Fellow 6). There was also some concern that the field was still highly stigmatized (see Table 1, 1.1).

Respondents felt that positions for researchers could arise from three arenas: academia, clinical settings, and government. Except in South Africa, academics and fellows stated that the primary focus of academia was teaching. They believed that there is good reason for this, given the dearth of clinical staff. Some fellows emphasised that their academic career tracks were limited to teaching and mentoring students; they were uncertain of the link between research and what they considered academic work, which primarily consisted of teaching (see Table 1, 1.2a–c). As mental health practitioners were already a scarce commodity and funding for research was limited, physician fellows especially could not envision how to build a research career in academia while continuing to practice:

*“I think [mental health clinicians’] workload is amazing and I can’t think that at the moment they have enough time to do research, let alone… to write because the pressures that they have in terms of clinical work is just overwhelming.”* (academic leader 1, Malawi; see also Table 1, 1.3)

Finally, a number of respondents highlighted that universities often lacked a route to allow clinicians to be part-time researchers within the university system (see Table 1, 1.4).

*“So, if you really want to improve efficiency in research…, then you have to come up with a clear [career] pathway for [clinical academics] to go into.”* (academic leader 1, Ethiopia)’

Some academic respondents, however, acknowledged that their universities were slowly recognising the importance of a balance between teaching and conducting locally relevant research. Similarly, a respondent from a funding organisation spoke about the need for a critical mass of researchers to sustain career structures:

“*So, you know what the pyramid is like for research: it’s this huge attrition before you even get to PhD or MSc…. But fundamentally there are not enough people working at a senior level.”* (head of section, International funding agency 4)

When international funders spoke about building research capacity, many saw the PhD as a first step in a longer trajectory. A PhD would be followed by post-doctoral study, which would lead to an opportunity to lead smaller research, and eventually to larger research studies. Conversely, fellows spoke about never having considered a post-doctoral fellowship before learning about it through AMARI, and in-country academics were still scrambling to provide strong PhD programs. While the South African university had structures in place to support post-doctoral research, in other countries, “the resource limitation was so huge, it makes this post-doc a luxury.” (academic leader 1, Ethiopia). While the Malawian university had begun to develop their post-doctoral programme, the university currently funded just six post-docs across all disciplines.

Interviewers asked academics, policymakers, service users, and fellows if there were career options for researchers in government. A number of academic and policy respondents believed this was possible and would increase government’s ability to use research more proactively. Others expressed concern that bureaucratic effects within government settings would hinder their ability to conduct rigorous research, with one fellow stating*, “Once they get into ministry staff, they become the administrator, not the researcher.”* (AMARI Fellow 3). Some fellows also worried that funding for research positions could not happen without changes at the policy level. These respondents felt that, at the outset, what was needed was better collaboration between researchers and policymakers (see Table 1, 1.5a, b)

### Research skills

Many respondents acknowledged that mental health research capacity was growing, in part due to the relatively new appreciation of mental health as a public health problem. Funders also acknowledged that competencies had improved. In Ethiopia, the department of psychiatry was seen as one department with the greatest number of publications, listed in the top 10 (out of 400): “*That is an indicator that there is a lot of research going on*.” (academic leader 1, Ethiopia). However, most respondents acknowledged that capacity still needed further development.

Many respondents highlighted the important role of mentors in building research capacity (see Table 1, 2.1). Fellows echoed the importance of mentoring: *“Mentoring is about helping me spread my wings, not following in a supervisor’s footsteps.”* (AMARI Fellow 8, see also Table 1, 2.2). A few academics appreciated that mentoring required a particular mindset and was not an automatic skill for senior researchers (Table 1, 2.3, 2.4). They also noted a dearth of senior researchers to be mentors, and one respondent described the extraordinary burden of being a senior researcher in a LMIC:

*“It’s not just [that] l am giving you two weeks of stats training or a month of research methodology training. l have a role to help create and build an infrastructure within which you can embed your research, develop your research, grow your research.”* (academic leader 2, South Africa)

Some academics and one international funder suggested that one way of building a larger pool of in-country experts who could support up-and-coming researchers was to reach out to other disciplines. As one academic stated:

*“The Department of Psychiatry has a group of researchers from different disciplines working together and we have seen some important publications and developments and improvements.”* (academic leader 3, Ethiopia)

Fellows also reiterated the importance of integrating their work into other disciplines, because they felt it improved their funding opportunities (Table 1, 2.5) and made them feel less isolated. They talked about the value of networking, which leads to greater exposure to senior researchers who are potential mentors (see Table 1, 2.6a, b). International funders also discussed networking and shared how they put in this extra support for creating links:

*“And then I would say one of the most important things we do is we link researchers who have never had an [organisation] grant, with [individuals] who have… that experience and that guidance… is really invaluable.”* (head of section, International funding agency 5)

### Funding

Many in-country academics talked about the importance of funding for research careers:

*“l have had more than one post*-*doc come to me [who is] highly skilled, highly motivated, and say they think they need to stop doing research because they can’t afford [it], because their families are expecting them to fund them.”* (academic leader 2, South Africa)

International funding organisations also emphasized the importance of funding protected time to develop research:

*“We do have …an award…that buys their time to do research. Because that is invaluable, it’s precious. It’s really hard to do really strong, rigorous …quality research on nights and weekends.”* (head of section, International funding agency) 5

International funders, however, highlighted that there is increasing funding for global mental health. As one funder shared,

*“[mental health] shot up the priority rankings within [our organisation] in the last 18* *months…So… there would be a hell of a lot more research money floating around.”* (head of section, International funding agency 4)

Another international funder noted that applications to conduct mental health research from LMICs were still too few overall. This was, in part, attributed to challenges with research support and grant management in LMICs. One funder observed:

*“But [our funding and reporting] process … is very involved. And domestic [local, in*-*country] researchers have generally an office of sponsored programs or grants management, that helps them. That doesn’t often occur in low*- *and middle*-*income countries. Researchers there have to do all of those pieces themselves.”* (head of section, International funding agency 5; see also Table 1, 3.1)

This led to funders having concerns about reporting when they awarded grants to LMICs:

*“Obviously, [my organisation] is accountable to this country’s taxpayer, and we, have huge reporting requirements… so it’s kind of a cyclical problem, because when you’ve received lots [of grants], then you have the experience of reporting and know what donors want.”* (programme officer, International funding agency 2; see also Table 1, 4.1)

While all funders recognised the importance of funding research in LMICs, they employed different approaches for grant administration and management. Some international funders emphasised a more top down approach where capacity was ‘injected’ into countries. For example, one funder required that an institution from a high-income country lead grants in collaboration with LMIC institutions:

*“We mandate that they are partnered with institutions in LMICs. I think part of the reason is that in LMICs sometimes they don’t have the … financial rigour. So, we feel comfortable giving large amounts of money to northern institutions [where] we know that they have … those financial systems that we need.”* (programme officer, International funding agency 2)

Another donor’s approach promoted existing capacity that needed to be “pulled out” or developed:

*“I think there’s sort of two components of the way we run our programme that have been really important. One is the fact that we require low, middle income country institutions to be the lead grant holder.”* (programme officer, International funding agency 1)

Other funders talked about the need to build capacity of LMIC academics to submit successful grant applications. This support included recruiting experienced researchers as mentors, inviting applicants to discuss ideas ahead of writing their proposal, and setting up collaborative research hubs.

### Low and middle-income country research commitment

While international funders understood their own critical role in funding research and building research capacity in LMIC, they felt there needed to be more of a push from in-country governments.

*“I think that anybody who does research in a low*- *and middle*-*income country has responsibility. …We attach research capacity*-*building to our research studies. But it’s not a substitute for the country committing to building up its own research infrastructure.”* (head of section, International funding agency 5; see also Table 1, 4.1, 4.2, 4.3)

Their emphasis was that research and research capacity needed to be viewed as integral to a country’s development:

“*And recognising that research is an important part in the overall development agenda. So, it is not something that is luxurious, that can only be invested in when …you reach a certain development threshold.”* (programme officer, International funding agency 6)

In Ethiopia, some academic respondents expressed frustration with a research culture that waited for others to come to them with resources:

*“So, from this side we have bigger [problems]: even when we set priority areas, we are not going forward to look for support or collaborative research teams outside.”* (academic leader 3, Ethiopia)

Academics and policy makers across the four countries echoed this need for government to ‘step up’ and appreciate their role in building local research capacity and deepen their commitment:

“*l would say the greatest culprit is the ministry for failure to provide the resources for people to do mental health…research.”* (researcher in policy^1^; see also Table 1, 4.4, 4.5)

In two countries, Ethiopia and Zimbabwe, respondents shared that governments have reacted to the need to enhance research capacity with a push to sizeably increase the number of PhD graduates. While academics in both countries appreciated the need for more high-level expertise, there was also a concern that the rapidity of this exponential increase would provide numbers without the requisite quality:

*“[our university was] mandated to take 1000 PhDs… You just need to train so that we will have faculty for all these universities… but for me, PhD level training is high level training… [is someone] who actually thinks, reflects.”* (academic leader 1, Ethiopia)

*“…so if you just recruit people and give them PhDs, that’s not going to help in terms of quality.”* (academic leader 5, Ethiopia; see also Table 1, 4.6)

### Advocacy: bridging the gap between research and policy?

Across the four countries and the funding community, respondents recognised that the legitimacy of mental health as a priority predominantly lay with government. They also agreed that ultimately, the role of research was to guide national policy (see Table 1, 5.1). Policymakers and academics frequently discussed the importance of having named government departments, posts, plans, or strategies for mental health; if there was not a policy or department that was dedicated to mental health, action would not happen.

To raise the profile of mental health, researchers discussed how they could best advocate to government. All types of respondents, including service users, stated that academic language needed to be simplified (Table 1, 5.2a, b). One service user offered to translate academic work into language that would be easier for policymakers to interpret. Policymakers defined the emphasis on publications and academic language as an indication that academics were not ‘practical’ in the way they considered themselves to be:

“*Academics they like to publish: ‘I…am publishing in so many peer review journals, first author, second author…’ That doesn’t mean anything to a policy maker. ….We have practical concerns: how is it going to help me reduce the problems that we are having in the hospital?*” (policy maker 4, Malawi)

However, academics stated that their research priorities were equally focussed on engaging with practical solutions to improve mental health outcomes in their respective countries.

Policymakers felt that an important advocacy strategy was for researchers to be present at their meetings. In most countries, the equivalent of a technical working group was a good place to start:

“*For example, if you do something on substance abuse, at least the mental health department for the Ministry of Health should have an idea of what is happening. And, if possible, be part of it so that the research findings [can] easily reach up to the technical working group.*” (MOH researcher 1, Malawi).

Many policy makers felt that academics did not take advantage of these opportunities.

*“The sad thing is that I find that most of the academics don’t realise that there are these technical working committees, but these are committees where people with a specific expertise can attend, present their findings, … their theories, and … whatever they have to shape policy.”* (academic leader 1, Malawi).

Moreover, respondents across the spectrum strongly agreed on the importance of involving policy makers at the outset of the research development (see Table 1, 5.3). Service users concurred and also asked for more involvement in the research process.

Many respondents stated that the onus for knowledge translation was predominantly on the researcher. However, one policymaker from Malawi described how a programme that involved training and mentorship on evidence-based policy formation improved their ability to appreciate research findings. Close to 40 middle-level managers from Parliament and the Ministry of Health were trained on how to translate research findings into policy briefs and then mentored for 1 year: *“Now, after that, we were able to develop guidelines for evidence use here in Malawi.”* (policy maker 4, Malawi). In addition, policy makers interviewed in Malawi felt that the current review of the mental health policy was more informed by research data (see Table 1, 5.4). They also appreciated how research data could provide strong guidance in developing policy in resource limited settings (see Table 1, 5.5).

Several international funding organisations equated research success with influencing policy, and there were a number of examples of this in the transcripts from in-country researchers. In Malawi, data pertaining to depression in youth supported the inclusion of mental health into the school health curriculum (Table 1, 5.6). In Zimbabwe, research data influenced alcohol and tobacco policies (see Table 1, 5.7). In Ethiopia, research data identifying epilepsy as the most common disorder seen at primary health care centres spurred the ministry to increase supplies of necessary drugs, train health professionals, and improve their tracking system (See Table 1, 5.8).

## Discussion

To our knowledge, this is the first study to investigate how to develop sustainable career tracks for mental health researchers in Africa. Our qualitative interviews with early-career researchers, policymakers, service users, African academics, and international funders revealed that while *all* stakeholders want more high-impact LMIC researchers, there are no clear pathways for developing their careers in African countries. While respondents highlighted the importance of the provision of quality research training programmes, there has been less support for the infrastructure that enables high-quality research: grants administration, mentorship, university leadership, research culture, and open communication between policymakers and researchers.

Respondents showed enthusiasm for research and believed that it is important, but they saw no clear replicable track to a research career within their universities or ministries of health. While there are selected examples of research excellence and successful African researchers, these academics were the exceptions, and respondents did not indicate that they knew how to replicate such trajectories. More often, both fellows and senior researchers noted the lack of senior mental health researchers to provide mentorship, for fellows and faculty alike, a finding consistent with prior studies [19]. Senior researchers in LMICs faced the burden of training clinicians and researchers, conducting their own research, and, often, fulfilling clinical duties, all without the administrative support provided to HIC clinician-researchers [18, 29]. Beyond careers in academia, all respondents agreed that there could be a role for doctorate-level researchers in ministries of health and wanted more linkages between research and government. There were, however, even fewer career pathways for this than there were in academia, in part because of lack of funding or dedicated offices for mental health at a government level. Many respondents identified that their universities and ministries of health did not have a ‘research culture’ in part because of the pressure to train clinicians and Master’s-level students. This weak ‘research culture’ may also be reflected in differences in the steps to a research career: many LMIC respondents saw a PhD as the end of research training with post-doctoral fellowships as ‘luxuries,’ while HIC funders considered PhDs as the first step, recognising that post-docs and early-career grants should naturally follow.

A clear tension emerged in the funding community between those who prioritized giving grants directly to LMIC institutions and those who wanted to support research in LMICs, but expressed concerns regarding their capacity to manage grants. Those funders typically supported partnerships between HIC institutions who took responsibility for the funds and who partnered with African institutions. While both funders and African-based respondents highlighted the challenges that African institutions face with grant administration and management, these challenges are not insurmountable. One funder described how they prioritized directly funding African institutions. AMARI and PRIME are both examples of successful programs where African universities were responsible for the distribution of funds and funder reports. In order to promote this paradigm shift, some study participants argued that funders can also support African institutions to develop stronger administrative capacity, which has begun to be developed as part of some recent mental health capacity building efforts [15]. HIC collaborators can partner with African institutions not only on research projects, but also on research administration and management, placing the administrative centre in African countries. Moreover, grant writers from African institutions can request funding for capacity building in administration and grant management.

Researchers, service users, and policymakers had similar goals to improve mental health services, but continued to articulate them differently. Policymakers were open to translating research into policy, especially if they had additional training, and shared some examples of this. Academics wanted their research to inform policy and believed that policymakers drove the mental health agenda [30, 31]. However, all groups highlighted that researchers did not present findings in lay language and were often absent from policy meetings or conversations, findings that echo prior work on the disconnect between the many voices advocating for global mental health [32]. The AMARI fellows, relative to more senior researchers, more often articulated specific ideas of how to bring their research to the attention of policymakers, perhaps because of the training on advocacy they have received through AMARI, which included training on how to engage with policymakers and the general public, creating policy briefs and lay summaries.

There are a number of ways to shift the balance of power to promote the careers of LMIC researchers. First, while there has been impressive recent investment in building research capacity, this must continue, both as stand-alone research training initiatives, such as AMARI [21], IMHERZ [13, 15] and capacity-building integrated into larger studies, such as PRIME, EMERALD, and the US National Institutes of Health hubs such as AFFIRM and PaM-D [4, 16, 17, 20]. Research capacity building initiatives should evaluate themselves based, in part, on whether the individuals who are trained go on to have research careers focussed on issues relevant to their home countries [4, 33, 34]. Second, these capacity-building interventions should include more training on communication with policymakers and should encourage researchers to involve policymakers early in their research process [25, 29]. In parallel, we need more support for training policy makers within ministries of health to use existing research evidence to develop evidence-based policy and formulate research questions [35]. Third, we echo prior academics [32, 36] in calling for funders to seek to develop the capacity of LMIC institutions to manage their grants, allowing more grants to be given directly to LMIC institutions and more interventions to be developed and tested locally. Fourth, LMIC researchers should feel empowered to take on the roles of first, corresponding, and senior authorship, with HIC researchers playing more supporting and coaching roles if required [4, 34]. We acknowledge that this would involve a fundamental shift in how HIC universities promote and incentivise researchers working in LMICs. However, to quote Abimbola, “to make global health truly global is to make global health truly local.” [37]. Fifth, research and clinical workforces must be developed in tandem. While recognising that the shortage of mental health clinicians in LMICs remains severe [8, 38, 39], research, and in particular the involvement of clinicians in research, is also important for health systems strengthening [13, 24]. Furthermore, clinical departments that are engaged in globally-recognised research may attract clinicians into typically-stigmatised specialties. More research funding going to LMICs will allow clinicians to more easily divide their time between research, teaching, and clinical work, helping develop a “research culture” at LMIC universities. One concrete way to support the capacity of more senior researchers and clinicians to teach and mentor might be to invest in increased administrative support for these individuals. LMIC universities, including African universities, should also develop policies that would allow clinicians who receive grants to ‘buy out’ clinical or teaching time for research. All of these aspects can draw on the experiences from other fields such as HIV or agriculture, where there has been greater progress on improving the research capacity of LMIC researchers.

This study has several limitations. First, half of the international funders did not reply or did not feel as if they were appropriate to interview. We did, however, get a depth of perspectives from funders as those who were most invested in global mental health funding were included. Additionally, by using purposive and chain referral sampling, we were able to develop an overall sample that was both deep and broad. No participants in the remainder of the sample declined to be interviewed. Second, the sample was limited to sub-Saharan Africa, and results cannot necessarily be generalised to all LMICs, as other regions (South America, Asia) may have better developed academic infrastructure and may face different challenges. Third, the results were not triangulated with university or national policy documents or with what grant funders are offering. However, we were interested in better understanding how LMIC academics perceive these policy documents, as that perception is what shapes how they interact with them, rather than what the policy documents actually state. Fourth, interviews took place within an existing capacity-building project, which may have made early-career researchers prone to desirability bias in their responses, but they were nevertheless important to include in the sample, as they are the focus of the project. To counteract this potential bias, we had an independent researcher not involved in AMARI conduct the interviews with fellows. Finally, while service users echoed similar points, their voices are not as represented in this study. Moving forward, we advocate for research that looks more specifically at service users and their organizations.

## Conclusion

To strengthen mental health systems in Africa, there is great need for locally developed research to guide practice and policy. To quote Abimbola, “to make global health truly global is to make global health truly local.” (Abimbola [37]). Within this broader frame, this study has focused on a neglected component of mental health research and advocacy: sustainable career tracks for African researchers. Given the critical component of mental health in African countries, socio-culturally appropriate and contextually relevant interventions need to be developed and implemented by researchers from those countries who best understand their own context. To promote career pathways for these researchers, there must be a paradigm shift to place the control of resources, study design, and authorship in large research projects in Africa and other LMICs, with HIC institutions in more supporting roles. This will require major changes in the way that HIC investigators and institutions conduct global health research to include more equitable ways of partnering with LMIC institutions.

Suggestion box: next steps International funders have a central role to play in shifting the centre of power in global mental health research. They should:

- Support the development of offices of grant management and administration at LMIC universities and provide training in grant administration and financial management (as has been done in other fields, such as support for building and staffing national HIV laboratories in Africa)
- Prioritize giving grants directly to LMIC institutions, either without HIC institutions on the grant or with HIC institutions as supporting partners.
- Ensure that research capacity-building initiatives includes training in grant writing and funding for post-doctoral and early faculty positions (beyond degree programs). This could help early career researchers have more time for research, away from clinical or teaching responsibilities.

Universities in LMIC should work toward:

- Creating contracts that allow clinicians to split their time between teaching, research, and clinical work. This would include giving them the option to “buy out” their clinical and teaching time if they receive research grants.
- Investing in developing infrastructure in grant writing, administration, and management so that university researchers would be better positioned to apply for and receive large grants. This could start with creating a position for someone who researches available grants, publicizes these opportunities, and provides support for researchers who are interested in applying.
- Investing in training in mentorship and supervision for both senior and early career researchers and should have mechanisms to recognize excellence in mentorship and supervision when considering promotion.
- Integrating research into training from the undergraduate level, including into medical school curricula to help foster a research culture.
- Creating a network of African universities that can foster a regional dialogue and share best practices on how publishing and position in the author order fits into promotion. The researchers we interviewed expressed frustration about the current system at many universities, where they often did not feel incentivized to participate in high-quality, collaborative research.
- Improving available skills and resources, departments of psychiatry and psychology should collaborate with other departments, such as maternal health or infectious disease, on research.

## Supplementary information

**Additional file 1: Appendix A.** Background to the four institutions supported by AMARI.

**Additional file 2: Appendix B.** Interview Guides.

## Data Availability

Data are not publicly available as the information shared in the transcripts make it possible to identify the respondents. As such, this data is available from the corresponding author on reasonable request.

## Footnotes

1 Country name was removed to preserve anonymity.
