# Supplementary material for: “We need more big trees as well as the grass roots”: going beyond research capacity building to develop sustainable careers in mental health research in African countries
Source: Int J Ment Health Syst. 2020 Aug 14;14:66. doi: 10.1186/s13033-020-00388-1 (PMC7427069; doi:10.1186/s13033-020-00388-1)
Supplement: Supplementary file 1 — Additional file 1: Appendix A. Background to the four institutions supported by AMARI. [file 13033_2020_388_MOESM1_ESM.docx]

**Appendix A: Background to the four institutions supported by AMARI**

**Ethiopia: Addis Ababa University**

Addis Ababa University (AAU) is the oldest and largest university in Ethiopia. Established in 1950, it now has 52,000 students, with 70 undergraduate programmes, 221 Masters programmes, and 72 PhD programmes. The new strategic plan emphasises the need to increase the percentage of women faculty (from a baseline of 14% in 2015) and women PhD students (from a baseline of 10% in 2015).

The Department of Psychiatry was made a WHO collaborating Centre in Mental Health Research and Capacity-Building in 2018, in recognition of decades of mental health research with international impact. This includes the Butajira severe mental disorder outcome study, which is one of the few population-based epidemiological studies of severe mental disorder from a low-income country.

Within AAU, the Department of Psychiatry has one of the highest publication output and citation rates, with over 300 publications. The dept of psychiatry has collaborative relationships with a number of African, Asian, European, Canadian and American research institutions, including Kings College London, Harvard University, University of Toronto, University of Cape Town, University of KwaZulu Natal, PHFI, University of Ibadan, University of Zimbabwe, and University of Malawi. The Department of Psychiatry is staffed by 17 full time professors and 2 adjunct associate professors. Fifteen of them are psychiatrists and 2 are clinical psychologists. Six of the staff have PhDs.

**Malawi: College of Medicine**

The College of Medicine (CoM), based in Blantyre with a satellite campus in Lilongwe, was established in 1991 as a constituent college within the University of Malawi (established in 1964). The college attracts Malawian and international students and currently offers ten undergraduate courses: a Bachelor of Medicine and Surgery (MBBS), BSc in Internal Medicine, BSc in Paediatrics and Child Health, BSc in Obstetrics and Gynaecology, Bachelor of Trauma and Orthopaedics, BSc in Health Management, BSc in Medical Laboratory Science (Hons), BSc in Pharmacy (Hons), BSc in Physiotherapy (Hons).

Its signature program remains the Bachelor of Medicine and Surgery (MBBS) degree – a 5-year program in clinical medicine, and to date, the only programme training doctors in Malawi. Candidates usually enter college following a one-year premedical training or after completing a two-year preparatory science course.

The CoM offers Masters of Medicine (MMed) degree programs in various clinical medical specialties such as Internal Medicine; Surgery; Pediatrics; Obstetrics and Gynecology; Ear, Nose and Throat; Psychiatry and Ophthalmology and also diploma and degree programs in Public Health (PGDPH and MPH). It also offers research-based masters and doctorate degree programs (MPhil and PhDs) in health-related fields in which the College of Medicine has strategic interests.

The College consists of 13 departments – i) Anaesthesia, ii) Basic Medical Sciences, iii) Family Medicine, iv) Information Technology, v) Internal Medicine, vi) Medical Laboratory Sciences, vii) Mental Health, viii) Obstetrics and Gynaecology, ix) Paediatrics and Child Health, x) Pharmacy, Physiotherapy and Surgery.

Mental Health Training at the College of Medicine:

The Department of Mental Health is the smallest department in the College of Medicine. Its currently consists of the head of department Dr Rob Stewart (MRCPsych, PhD), a UK trained psychiatrist and senior lecturer, clinical psychologist Dr Chiwoza Bandawe (MA (ClinPsych), PhD (SA); Assoc Prof, Senior Lecturer) and two Malawi/South African trained psychiatrists/lecturers, Dr Kazione Kulisewa and Dr Olive Liwimbi. At present, four Malawian doctors are conducting specialist training in Mental Health/Psychiatry (MMED) in the department and the aligned teaching hospitals. The department is supported by volunteer honorary clinical tutors (mainly from the UK) who join the department for teaching periods ranging from 3 weeks to 6 months.

The undergraduate mental health course at the CoM is held in year 4. We currently run four six-week long rotations in general adult mental health for 100-120 students per academic year. We also run a one-week child and adolescent mental health teaching block twice during an academic year. We encourage students to do their year 3 research projects in the field of mental health and offer supervision.

The department is supported by a Scottish Government grant via the Scotland Malawi Mental Health Education Project, which funds the training of the four MMEDs in Psychiatry.

**South Africa: The University of Cape Town**

The University of Cape Town (UCT) is South Africa's oldest public research university located in Cape Town, South Africa. UCT is listed among the top 171 universities globally, according to the Times Higher Education World University Rankings, and is currently the highest-ranked African university. In 2018, UCT’s Faculty of Health Sciences (FHS) was ranked in the top 70 universities for clinical, pre-clinical, and health sciences in the world. The FHS is the oldest medical school in sub-Saharan Africa and has built a reputation for distinction in teaching, training, service and cutting-edge research. The FHS campus extends from its main teaching hospitals in Cape Town to a range of secondary hospitals and primary health care clinics throughout, and beyond the Cape Peninsula. Some famous breakthroughs in health care, including the first successful heart transplant in the world in 1967 and the pioneering research that led to the development of the CAT scanner (and a Nobel Prize), placed the faculty on the map as a world-class facility in sophisticated, tertiary medicine. At the same time, the faculty is driven by a strong primary health care vision relevant to Africa, with much current research involving prevention and implementation science at a primary care level. In pursuit of its goals, the faculty’s key strategies and objectives are aligned with the broader goals of the University and with mandates from the National Departments of Higher Education, Health, and Science and Technology, as well as local partners in the South African Medical Research Council, National Research Foundation, National Health and Laboratory Services, and the Health Department of the Western Cape Provincial Government.

**Department of Psychiatry and Mental Health**. A significant amount of the department’s research is of an applied nature and is paralleled by involvement in policy initiatives at national and international levels. A major new initiative of the Department in 2010 was the launch of the Alan J. Flisher Centre for Public Mental Health (CPMH) dedicated to the memory of Prof. Alan J. Flisher, a pioneer of African public mental health.

The main objectives of the CPMH are (1) to undertake high quality research in the areas of public mental health, mental health policy, services, legislation and human rights; (2) to develop capacity in Africa for public mental health research, mental health policy, planning and legislation, through accredited post-graduate academic teaching programmes; (3) to provide advisory services to provincial and national government in South Africa, governments in other African countries, and international health and development agencies, with the goal of strengthening mental health policy and systems; and (4) to advocate for the inclusion of mental health on health policy and development agendas in Africa// to conduct high quality public mental health research, and to use evidence for teaching, consultancy and advocacy to promote mental health in Africa. From UCT’s perspective, the work of the CPMH is aligned with several key strategic goals of the university, namely to conduct high quality inter-disciplinary research, expand the field of research in a particular area by raising large independent research grants, develop niche areas that respond to particular research challenges in sub-Saharan Africa, and conduct research that is socially responsive.

The CPMH employs a multi-disciplinary team dedicated to undertake high quality research in the areas of public mental health, mental health policy and services. We have 22 staff currently employed at our UCT office, with approximately 20 more employed by UCT in fieldwork or service sites in Cape Town, and over 100 employed in our partner institutions in the 8 countries in which we work. In addition to currently leading some of the largest mental health research grants in low and middle-income countries, significant achievements of the CPMH include accreditation as a WHO Collaborating Centre (2015), and accreditation as a UCT University Research Centre (2014).

**Zimbabwe: The University of Zimbabwe**

Established in 1955, the University of Zimbabwe is the oldest university in Zimbabwe and is involved in teaching, research, community service, and innovation. Located in the capital, Harare, the university offers degrees, diplomas and certificates in various disciplines which include arts, agriculture, law, medicine, social studies, science, engineering, education, commerce, and veterinary sciences. All its programmes are accredited by the Zimbabwe Council for Higher Education and other professional bodies in medicine, law, engineering, accountancy, social work and veterinary science.

The Department of Psychiatry is housed within the College of Health Sciences. The department enrolled its first students in 1981. At its inception, the Department of Psychiatry had one psychiatrist. Currently the Department includes six of the 15 psychiatrists in Zimbabwe. The staff compliment for the Department of Psychiatry is 12 which includes one adjunct associate professor. In addition to the six psychiatrists, there are three clinical psychologists and three nurses. Two of the staff have PhDs. In 2011, the Department of Psychiatry was awarded the Medical Education Partnership Initiative linked award in Mental Health in Africa “Improving Mental Health Education and Research in Zimbabwe” (IMHERZ) which focused on building the capacity of mental health research. The Department of Psychiatry has spearheaded the establishment of two child and adolescent mental health clinics in Harare. The department teaches the following programmes: i) Diploma in Mental Health; ii) M.Med in Psychiatry; and, iii) DPhils in Psychiatry. Students studying for a bachelor’s degree in medicine (MBChB) are offered courses from the department in their first, second, and fourth years. Students studying for a bachelor’s degree in dental surgery are offered courses from the department in their first and second years
