# Supplementary material for: “We need more big trees as well as the grass roots”: going beyond research capacity building to develop sustainable careers in mental health research in African countries
Source: Int J Ment Health Syst. 2020 Aug 14;14:66. doi: 10.1186/s13033-020-00388-1 (PMC7427069; doi:10.1186/s13033-020-00388-1)
Supplement: Supplementary file 2 — Additional file 2: Appendix B. Interview Guides. [file 13033_2020_388_MOESM2_ESM.docx]

**APPENDIX B: INTERVIEW GUIDES**

**A system level analysis of career opportunities, pathways and funding priorities for mental health, neurological, and substance use (MNS) research in countries taking part in the [name of the programme]**

Separate interview schedules have been identified for: a) university level; b) funding bodies; 3) ministry of health; and 4) service user representatives.

1. **University level:**

We would like to understand more about the career pathways and the research environment for MNS (mental health, neurological, and substance use) researchers in this institution:

**A. Can you tell me about MNS research at your institution?**

-> What do you think about the current strategy for MNS research at your institution?

**->** What MNS research is currently being done at your institution?

-> How does MNS research fit in with your other research priorities?

**B. What are the priorities for MNS research specifically?**

-> probe for differing views between the department and University leadership.

**C. What are the current challenges in building up sustainable MNS research?**

-> Can you tell me about examples of research projects that have not gone well?

-> Or an example of when someone wanted to do MNS research and was not able to?

**D. What funding is available for MNS research?**

Ask only if the respondent does not explain these issues in the course of answering the first question:

-> What support does your institution provide for people seeking research funding?

-> What would make it easier/possible for you to seek research funding?

-> What are the barriers to applying for or receiving research funding?

-> Probe for specific examples of institutions/groups/individuals got funding – what worked well in these cases?

**E. What are the current structures for career pathways/development/progression at your institution?**

What does it look like to have a career in mental health research at your institution/in your country?

*Strengths:* Within the current structures, what is working well?

-> What has facilitated things going well? Probe for specific examples of effective career trajectories.

*Weaknesses:* Difficulties within the current structures are?

-> Incentives/values/time burden/lack of role model/clinical responsibilities / institutional culture / individuals institution-? tensions / office space…

*Opportunities:* Are there examples of departments where this worked well? What made it work well? Are there examples of research careers outside of academia?

-> What could make a difference for MNS research careers?

*Threats:* What are the problems you envisage in creating/sustaining the structures for career development in MNS research?

- How could trainees become independent MH researchers? What would need to change?
- What are the tensions between clinical, teaching and research responsibilities for MNS researchers in your institution?

**F. To what extent do researchers at your institution typically transition between levels of training? For instance, between PhD and post-doctoral level.**

-> Can you tell me about a situation in which someone has transitioned between these roles?

**G. What do you envisage is/will be the role of senior researchers at [your university]?**

-> What should they be doing?

-> What would it mean for your institution to invest in senior leadership?

-> Are there financial resources available to support this?

**H. In terms of career progression and research: how do faculty members get promotion?**

-> How does research play into this? How important is it for researchers to bring in grants? How are they rewarded for obtaining grants?

**I. What is the value of having highly qualified researchers in MNS in [this country]?**

-> Where should they be employed/how can they contribute? Who should they be working for?

**J. How do researchers relate to health policy and planning in [this country]?**

-> How do you envision them becoming more or less involved with evidence-based health policy and planning?

**K. How is a clinical academic different from a clinician?**

-> What are the structures to link clinical academics in [this university]?

**L. How do you envisage the future of the [programme name] trained researchers at [this university] in [this country]?**

1. **Funding bodies:**

We would like to understand more about your organisation’s ideas regarding funding of clinical research.

**A. Which health or disease areas are most important to fund?**

- Why are these the most important areas?
- What kind of research is important?
- List areas and get them to rate from 1-10 – (policies, health systems, health services, medication trials, molecular/genetics, intervention studies, epidemiological studies)
- Within MH – what kind of research is important? – (policies, health systems, health services, medication trials, molecular/genetics, intervention studies, epidemiological studies, psychological therapies, involving traditional healers, mental health and culture,…)

**B. What do you think about funding MNS research?**

- Probe about why the respondent values/does not value MNS research.
- Probe about what areas of MNS research they think are important

**C. How much priority should be given to MNS research in [this country]? Why?**

**D. Can you give me an example of when MNS research received financial support? What happened?**

**E. Why has your organisation invested or not invested in MNS research in the past?**

**F. Within the area of NCD, why have developmental organisations not focussed on MH research?**

**G. How does MNS research fit into research in other areas of health care research?**

-> How do you see the opportunities for linking MNS research funding with funding for other areas eg HIV, NCDs, MCH?

**H. If you were to develop an agenda for funding MNS research in [this country] how would you go about establishing the agenda:**

- **Whom would you consult?**
- **How would you set priorities?**
- **Where would the funding come from?**

**I. What opportunities are available for linking local (national) funding for MNS research with other bigger international funders?**

-> Probe about whether this has happened for other disease conditions

1. **Ministry of Health**

**A. Can you tell me about MNS (mental health, neurological, and substance use)** **research in [this country]?**

**B. What do you think are the priorities in MNS research in [this country]?**

-> Why are these the most important areas?

-> List of areas and get them to rate from 1-10 (with 10 rating as most important)? – policies, health systems, health services, psychological interventions, medication trials, molecular/genetics, studies to quantify MH, traditional healers, CAMHS, MH & Culture,…

**C. How does MNS research fit into your overall public health strategy?**

-> From the point of view of your current policy priorities in your country, what are the most relevant research questions for MNS?

**D. Can you give me an example of a time when MNS research (or research in another area) impacted policy or planning?**

-> Probe about this experience.

**E. In general, how has research been relevant in informing health strategies in [this country]?**

**F. Are people able to build careers in mental health (MNS) research in [this country]?**

-> Can you give me an example of a mental health research career?

**G. How well do researchers currently communicate their work in MNS research to you? To the general public?**

-> Can you give me an example of when this worked well?

**H. How can researchers in [this country] better connect with policymakers?**

**I. What needs to change on the side of the academics to strengthen MNS research in [this country]?**

**J. What needs to change on the side of the ministry to strengthen MNS research in [this country]**

-> Probe about employing more researchers at the MoH – is this feasible? What would make it feasible?

1. **Service User Representatives**

**A. What have your interactions with mental health care in [this country] been like?**

**B. What have you found most frustrating/difficult about mental health care in [this country]?**

**C. What do you think about mental health research? What is it? Is it important?**

**D. If we were to work on studying one area of mental health care, what do you think that we should study? Why?**

**E. Does your country/university system have people who do this work?**

**F. If people were to do mental health research in [this country], how would you like to be involved? What role do you want service users to play?**

**G. How well do researchers currently communicate their work in mental health research to you? To the general public? How should they communicate their work to you?**
